# *In vivo *expression of innate immunity markers in patients with *mycobacterium tuberculosis *infection

**DOI:** 10.1186/1471-2334-10-243

**Published:** 2010-08-18

**Authors:** Pantelis Constantoulakis, Eftihia Filiou, Nikoletta Rovina, George Chras, Aggeliki Hamhougia, Simona Karabela, Adamandia Sotiriou, Charis Roussos, Nikolaos Poulakis

**Affiliations:** 1 Locus Medicus, Molecular Pathology and Genetics Dept., Athens, Greece; 2 National and Kapodistrian University of Athens, Pulmonary Department, "Sotiria" District Chest Diseases Hospital; Athens, Greece; 3 Mathematics Dept., University of Athens; Athens, Greece; 4 National Center of Tuberculosis, Microbiology Dpt, "Sotiria" District Chest Diseases; Athens, Greece; 5 1st Pulmonary Department, "Sotiria" District Chest Diseases Hospital. Athens, Greece

## Abstract

**Background:**

Toll-like receptors (TLRs), Coronin-1 and Sp110 are essential factors for the containment of *Mycobacterium tuberculosis *infection. The purpose of this study was to investigate the *in vivo *expression of these molecules at different stages of the infection and uncover possible relationships between these markers and the state of the disease.

**Methods:**

Twenty-two patients with active tuberculosis, 15 close contacts of subjects with latent disease, 17 close contacts of subjects negative for mycobacterium antigens and 10 healthy, unrelated to patients, subjects were studied. Quantitative mRNA expression of Coronin-1, Sp110, TLRs-1,-2,-4 and -6 was analysed in total blood cells *vs *an endogenous house-keeping gene.

**Results:**

The mRNA expression of Coronin-1, Sp110 and TLR-2 was significantly higher in patients with active tuberculosis and subjects with latent disease compared to the uninfected ones. Positive linear correlation for the expression of those factors was only found in the infected populations.

**Conclusions:**

Our results suggest that the up-regulation of Coronin-1 and Sp110, through a pathway that also includes TLR-2 up-regulation may be involved in the process of tuberculous infection in humans. However, further studies are needed, in order to elucidate whether the selective upregulation of these factors in the infected patients could serve as a specific molecular marker of tuberculosis.

## Background

In the majority of patients, tuberculosis (TB) is typically found as an airborne disease due to droplet nuclei infection. In the immuno-competent host, lungs are most commonly affected in *M. Tuberculosis *(MTB) infection, with estimates of 80-87% of lung involvement in subjects with active disease. A similar estimate of 70-90% has also been observed in immuno-compromised hosts, such as those with human immuno-deficiency virus infection (HIV) [1]. A person in close contact with a smear-positive patient is at maximum risk of being infected. Studies have shown that the infection rate among close contacts ranges from 25-50%, even in the worst over-crowded and sub-standard conditions, while among those infected only a 10% will progress to disease [2]. Thus, a great portion of close contacts remains uninfected after exposure and is probably due to the host's innate immune system.

The effective antimicrobial action of monocytes/macrophages against MTB infection consists of a well-organized antimicrobial machinery, with a large armamentarium of cytotoxic molecules, including Toll-like receptors, phagosome-lysosome function and the recently described tuberculosis resistance genes that are considered integral parts of this mechanism [3,4].

Toll-like receptors (TLRs) are a family of proteins that play a key role in the innate immune response to infectious agents through their ability to discriminate conserved microbial structures, known as pathogen-associated molecular patterns (PAMPs), from self. TLRs recognition of PAMPs, such as lipopolysaccharide (LPS), initiates signal transduction through the NF-kB pathway. Nuclear translocation of NF-kB induces transcription of pro-inflammatory cytokine genes essential to mounting a protective immune response and host defence [5]. TLRs' stimulation in macrophages has been shown to promote phagosomal maturation via activation of p38 MAP kinase in a MyD88 dependent signalling pathway [6,7]. Pathogen associated molecular pattern signatures for pathogenic mycobacteria include, CpG DNA, 19 kDa lipoprotein, lipoarabidomannan and mannosylated phosphotidylinositol (PIM) [8]. TLRs have been reported to participate in the interaction of pathogenic mycobacteria or in their extracellular products in mice and humans. In particular, TLR-2, in association with TLR-1, TLR-6 and TLR-4, has been implicated as a receptor involved in the recognition of mycobacterial antigens and activation of macrophages and dendritic cells (DCs) [9]. Furthermore, TLRs are essential in the killing process of intracellular mycobacteria by human macrophages through the induction of the antimicrobial peptide cathelicidin [10]. In addition, it has recently been shown that TLRs' non-synonymous polymorphisms are significantly associated with tuberculosis disease in humans [11].

Pathogenic mycobacteria have evolved a unique strategy to survive intracellularly within macrophages. They modulate phagosome maturation to prevent the fusion of endosomes with lysosomes. The block of phagolysosome biogenesis has been proposed to play a central role in the pathogenesis of the mycobacterium [12]. The prolonged survival of *M. tuberculosis *within their mammalian host cells suggested that these pathogens, apart from producing virulence factors such as phosphatase SapM and protein kinase PknG, have evolved mechanisms to utilise host molecules for their own survival [3,13]. An analysis of the protein composition of mycobacterial phagosome showed the exclusive presence of a protein that was strongly retained on phagosomes harbouring live mycobacteria [13,14]. This protein, initially named TACO (for tryptophan-aspartatecontaining coat protein) is now referred as Coronin-1. Coronin-1 is a member of the WD repeatcontaining protein family of coronins. The founding member of this protein family was identified in the amoeba *Dictyostelium discoideum *and is involved in the regulation of actin-based dynamics [15].

In mammalian cells up to seven coronin isoforms have been identified and were thought to regulate F-actin-dependent cytoskeleton [16]. Recent data indicate that Coronin-1 is essential for the survival of live mycobacteria into monocytes/macrophages, because this protein arrests the maturation of phagosome to phagolysosome. Coronin-1 is specifically restricting phagosomes containing live mycobacteria from entering the lysosomal pathway by regulating a calcium- dependent signalling process, which activates the calcium-dependent phosphatase calcineurin. This enzyme is responsible for the blocking effect of phagosome-lysosome fusion. In the absence of Coronin-1, calcineurin cannot be activated which results in lysosomal transfer and death of internalized mycobacteria. Similar results were also retrieved with calcineurin blockers, such as cyclosporine A or FK506 [17]. The prolonged retention of Coronin-1 in phagosome during active infection has been attributed to a secreted mycobacterial lipoamide dehydrogenase C (LpdC), which retains Coronin-1 on the phagosomal membrane [18] and is produced only by live BCG and *M. tuberculosis*.

Also, recent data have shown that inbred mice expressing the *Ipr1 *(intracellular pathogen resistance 1) gene in the *sst1 *locus were protected against infection by *M. tuberculosis *and *M. bovis*. The up-regulation of *Ipr1 *after infection by *M. tuberculosis *limits the infection in tuberculosis resistant mice by switching the cell death pathway of the infected macrophages from necrosis to apoptosis [19]. Sp110 is the closest human homologue of *Ipr1 *gene and is located on chromosome 2 (2q37,1). Sp110 is a component of the nuclear body, a multi-protein complex assumed to participate in the regulation of gene transcription. This gene is very important for monocytes' differentiation, apoptosis and activation including the response to pathogens [20,21]. Sp110 inhibits vesicular stomatitis and influenza virus replication. It also confers resistance to human Foamy virus, while gene polymorphisms or mutations have been associated with susceptibility to hepatitis C, immunodeficiency virus and hepatic veno-occlusive disease [22,23]. In this respect, Sp110 may also have a role in human tuberculosis infection.

Studies conducted so far, support the idea that intracellular factors like Coronin-1, Sp110 and TLRs play a decisive role in the progression of TB infection, at least in experimental tuberculosis. Hence, it is possible, that the expression of these molecules somehow regulates the intracellular fate of *M. tuberculosis *through a complicated cascade of up- and down-regulations. However, studies regarding the expression of these intracellular molecules in humans are lacking. We hypothesized that during active infection, because of the turnover of the infected monocytes and the following haematogenous spread of the infection, these markers could be increased in patients with TB.

The purpose of this study was to investigate the expression of these molecules in peripheral blood mononuclear cells and to look for any possible correlations between their level of expression and the disease status. We utilized a real-time PCR assay to measure the expression of mRNA encoding Coronin-1, Sp110 and TLRs in fresh whole blood cells of TB patients, close contacts to patients that had latent infection (Quantiferon positive, QFT (+)), close contacts to patients with no latent infection (Quantiferon negative, QFT (-)) and a negative control population. QFT testing was performed according to the CDC and the literature recommendations in all circumstances in which the TST is currently used, including contact investigations, evaluation of recent immigrants, and sequential-testing surveillance programs for infection control (e.g., those for healthcare workers) [24-26].

We demonstrated striking changes in the expression of Coronin-1, Sp110 and TLR-2, between the TB-infected population (actively and latently infected) compared to the uninfected controls, whereas the levels of the other TLRs did not differ significantly. Our data suggest that Coronin-1, Sp110 and at least Toll-like receptor-2 molecules are involved in the infectious process of tuberculosis. However, their significance and their precise mechanism in human tuberculous infection deserve further investigation.

## Methods

### Patients and samples

Twenty two patients with pulmonary tuberculosis (TB) (group A) were recruited at the "Sotiria Hospital", Athens (Greece). Patients' characteristics are shown in Table 1. Sixteen patients had pulmonary cavitary disease and or parenchymal TB, four patients pulmonary TB and TB lymphadenitis and two patients had concomitantly pulmonary disease associated with TB pericarditis and TB pleural effusion, respectively. The diagnosis of tuberculosis was confirmed by smear and culture positive for *M. Tuberculosis *from the respective biological specimens.

**Table 1:** Characteristics of the patients with active TB (group A), of the close contacts with latent TB infection (group B), of the close contacts without latent TB infection (group C), and of the control subjects (group D)

Group (N)	Age (ys) **Mean **± **SD**	SEX M/F	QUANTIFERON Positive/Negative
A [22]	43 ± 3	19/3	18/4
B [15]	43 ± 5	12/3	15/0
C [17]	35 ± 3	9/8	0/17
D [10]	35 ± 6	5/5	0/10

Fifteen volunteers from the close contacts of patients, that were tested positive for Quantiferon-TB (QFT), after an extensive examination to exclude tuberculosis, were considered to have LTBI and constituted the group B. Seventeen volunteers from the close contacts, that were tested negative for Quantiferon-TB in two consecutive examinations (8-10 weeks apart from each other) were considered not infected (group C). QFT testing was used for diagnosis of LTBI because of its greater specificity compared to intrademal Mantoux test according to the recent CDC recommendations for the investigation of contacts with an infectious case of tuberculosis [27].

The exposure time for close contacts identification, i.e the average daily exposure in hours multiplied by the days of exposure, during the last trimester preceding the tuberculosis diagnosis was also estimated according these CDC guidelines [27]. This estimation of exposure has also been validated in studies correlating the time exposure to an index case with the performance of the interferon gamma releasing assays (IGRAS) test [28]. The mean value of the average exposure time for close contacts with latent TB infection (QFN positive) was 665.6 hour-days, while for the close contacts negative for latent TB infection (QFN negative) was 648.82 hour-days.

Finally, ten healthy volunteers with no exposure to TB, matched for the age and gender were included, after tested negative for QFT.

Informed consent was obtained from all patients, close contacts to patients and control subjects, and the study was approved by the Ethical Review Committee of "Sotiria Hospital".

### Blood collection, RNA isolation, quantitative RT-PCR

Whole blood (2.5-5 ml) was drawn before or within the first week of anti-TB treatment and was immediately processed to prevent unwanted changes in RNA profile. Total RNA was extracted from 10^6 ^human peripheral blood nucleated cells (PBMCs) using the QIAamp^® ^RNA Blood mini kit (QIAGEN, GmBH), following the manufacturer's instructions. The RNA samples were stored in 40 μl of RNAse free distilled water at -40°C. The relative expression of Coronin-1, Sp110, TLR-1, TLR-2, TLR-4 and TLR-6 mRNAs was evaluated by quantitative real time reverse transcription PCR with the one step RT-PCR kit Light Cycler^® ^RNA Master HybProbe (ROCHE). Transcripts of β_2 _microglubin were initially quantified as endogenous RNA of reference gene to normalize each sample. However, because of the previously described instability of the above mentioned mRNA during MTB infection [29,30], we repeated the experiments using the human acidic ribosomal protein (HuPO) as a reference gene, that has been reported to remain stable during tuberculosis infection [31,32]. Indeed, we noticed (data not shown) a significant difference in our results regarding at least two of the genes analyzed. The results presented in this study refer to the human acidic ribosomal protein HuPO as the control gene. Taqman primers & probe combinations used for TLRs were designed according to a previous publication [33]. TIB MOLBIOL Syntheselabor, GmbH designed and produced the primers and probes used for detection of β_2 _microglubin (forward primer: CCAgCAgAgAATggAAAgTC, reverse primer: gATgCTgCTTACATgTCTCg, probe FL: TTCTTCAgTAAgTCAACTTCAATgTCggA-FL, probe LC: LC640-ATgAAACCCAgACACATAgCAATTCAg--PH), Coronin-1 (forward primer:CAATCCggTACTTTgAgATCA, reverse primer: gCTTgTAgAACCTggCgA, probe FL: CTCCTTggAACTgAACATggAgAgA-FL, probe LC: LC640-AgTgCAggAAAggggCCTCg--PH), Sp110 (forward primer: TCAgAggAgATCATTgATggC, reverse primer: CACTTggAgCTTCTCTTggAT, probe FL: AAAgAggTCCCAgAAgACgCCT--FL, probe LC: LC640-gTACACCACgAAgggTCACACAAggg--PH) and HuPO mRNA (forward primer: gCTTCCTggAgggTgTC, reverse primer: CCAAgAAggCCTTgACCTT, probe FL: CgAgTCCTggCCTTgTCTgTggA-FL, probe LC: LC640-CggATTACACCTTCCCACTTgCTg--PH). All primers and probes were stored at a concentration of 20 μM and 10 μM, respectively. In β_2 _microglubin, Coronin-1 and Sp110 reactions, 0.5 μM of each forward and reverse primer as well as 0.2 μM of the probes were used per 20 μl reaction, while in Toll-like receptors reactions the concentration of primers and probe were 0.5 and 0.1 μM, respectively. The PCR conditions were as follows: 61°C for 20 min (RT), 95°C for 1 min (DENATURATION), 95°C for 3 sec, 54°C(50°C for SP110/HuPO and 48°C for coronin) for 12 sec, 72°C for 10 sec (45 cycles), 95°C for 0 sec, 50°C(48°C for SP110/HuPO and 46°C for coronin) for 30 sec, gradually up to 80°C for 0 sec (MELTING CURVE), 40°C for 30 sec (COOLING).

### Data analysis

The Wilcoxon test for the difference of dependent and the Mann-Whitney-Wilcoxon test for the difference of independent means of the variables examined were used for the analysis in the present study. Correlation between the expressions of the variables examined within the different groups was calculated according to Spearman cofactor for linear correlation. The analysis was contacted using SPSS program version 16.0 for Windows.

## Results

### Effect of tuberculosis infection on the expression of immunity markers

**Figure 1:**
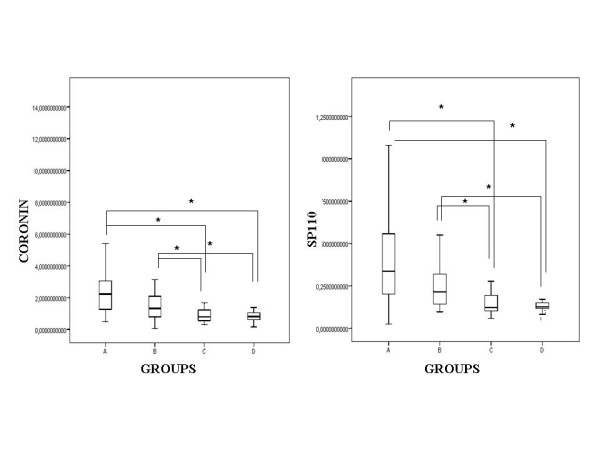
**Expression of mRNA encoding for Coronin-1 and Sp110 in fresh unstimulated whole blood in the various groups studied**. Data were normalized relative to the endogenous expression of the human acidic ribosomal protein HuPO. There was no significant difference in Coronin-1 and Sp110 expression (p > 0.05) between the patients' group (group A) and positive close contacts' group (group B). *p < 0.05, for Groups A and B as compared with the uninfected population (groups C and D).

**Figure 2:**
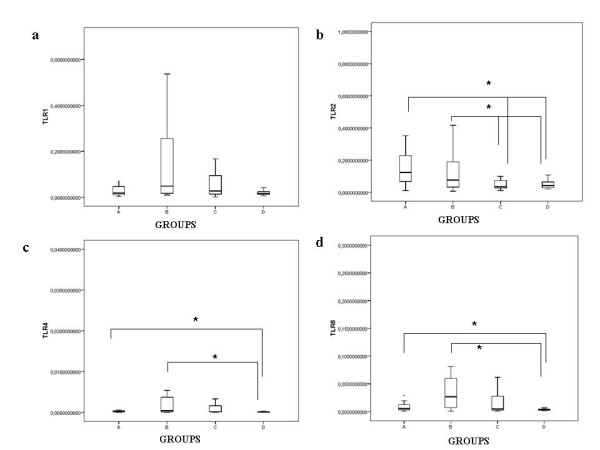
**TLR-1 (a), TLR-2 (b), TLR-4 (c), TLR-6 (d) in fresh unstimulated whole blood in the various groups studied**. Data were normalized relative to the endogenous expression of the human acidic ribosomal protein HuPO. There was no significant difference between groups A and B (p > 0.05) in a,b,c, and d. (a)*p < 0.05, for Groups A and B as compared with the uninfected population (groups C and D).

The comparison of mRNA levels (divided by the expression of the reference gene expression) of the different intracellular molecules examined between the various groups of our study is shown in Table 2 and separately for each factor in Figures 1, 2. Compared with the uninfected population (groups C and D), whole blood from patients (group A) and from latently infected subjects (group B) had significantly increased levels (*p < 0.05) *of mRNA encoding Coronin-1, Sp110 and TLR-2. In contrast, expression of Coronin-1, Sp110 and TLR-2 mRNAs were not significantly different between the patients (group A) and the close contacts with latent infection (group B). TLR-1 mRNA was not significantly increased in TB-patients (group A) *vs *uninfected subjects (group C and D), while TLR-4 and TLR-6 mRNAs were significantly increased (*p < 0.05) *only between actively infected patients (group A) and controls (group D).

**Table 2:** Primers and probes sequences for Real Time RT-PCR of β_2 _microglubin, Coronin-1, HuPO and SP110 mRNA

mRNA	Forward primer	Reverse primer	Probe FL	Probe
β_2_ microglubin	CCAgCAgAgAATggAAAgTC	gATgCTgCTTACATgTCTCg	TTCTTCAgTAAgTCAACTTCAATgTCggA-FL	LC640-ATgAAACCCAgACACATAgCAATTCAg--PH
Coronin-1	CAATCCggTACTTTgAgATCA	gCTTgTAgAACCTggCgA	CTCCTTggAACTgAACATggAgAgA-FL	LC640-AgTgCAggAAAggggCCTCg--PH
SP110	TCAgAggAgATCATTgATggC	CACTTggAgCTTCTCTTggAT	AAAgAggTCCCAgAAgACgCCT--FL	LC640-gTACACCACgAAgggTCACACAAggg--PH
HuPO	gCTTCCTggAgggTgTC	CCAAgAAggCCTTgACCTT	CgAgTCCTggCCTTgTCTgTggA-FL	LC640-CggATTACACCTTCCCACTTgCTg--PH

### Correlation between the markers' expression in PBMCs with the infection state

**Figure 3:**
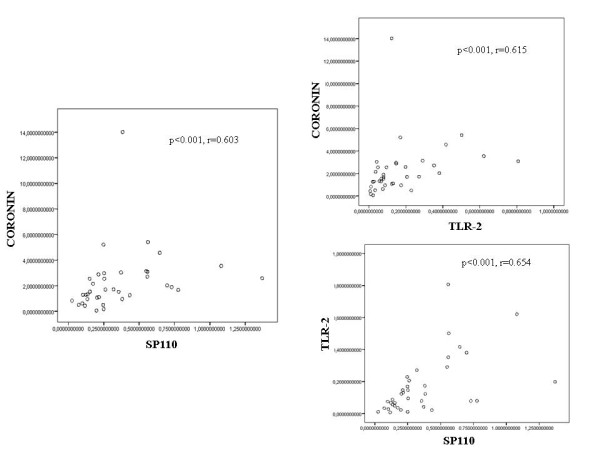
**Spearman correlations between the expression of Coronin-1 and Sp110 (a), Coronin-1 and TLR-2 (b), and TLR-2 and Sp110 (c) mRNA expression in the patients' (group A) and the positive close contacts (group B) groups**.

According to the Spearman cofactor for linear correlation, there was a positive correlation between Coronin-1 and Sp110 (r = 0.603), Coronin-1 and TLR-2 (r = 0.615) and between Sp110 and TLR-2 (r = 0.654) expression in the patients group (group A) and close contacts group with LTBI (group B) (Figure 3). By contrast, no significant correlation was found between the expression of these markers in the uninfected group of close contacts (group C) and in the control subjects (group D) (data not shown).

## Discussion

In this study, we observed an increased expression of TLR-2, Coronin-1, and Sp110 mRNA levels in PBMCs of patients with tuberculosis and in close contacts of patients that had latent tuberculosis infection. Furthermore, we found an increased expression of TLR-2 mRNA in patients with tuberculosis compared to the close contact group without LTBI, whereas there was no significant difference in the expression of TLR-2 mRNA between patients and close contacts with LTBI. These findings indicate that TLR-2 Coronin-1, and Sp110 are probably involved in the infection process of human tuberculosis.

TLRs have been reported to participate in the interactions of pathogenic mycobacteria or their extracellular products with mouse and human cells [34]. In particular, TLR-2 in association with TLR-1 and TLR-6, and TLR-4 have been implicated as receptors involved in the recognition of mycobacterial antigens and activation of macrophages and dendritic cells [35]. In a recent study, an increased expression of TLR-1, TLR-2, TLR-4 and TLR-6 has been reported at the mRNA level in peripheral blood mononuclear cells, but not in broncho-alveolar lavage cells from patients with tuberculosis compared to healthy controls [36]. The high expression of TLR-2 in PBMCs can be explained by increased circulating levels of pro-inflammatory cytokines or by circulating mycobacteria/mycobacterial components. Alternatively, it may simply represent the re-circulating monocytes from the pulmonary tuberculous granulomas, which are expressing TLR-1, TLR-2 and TLR-4, as previous studies have shown [37]. Accumulating data indicate that *Mycobacterium tuberculosis *expresses a large repertoire of TLR-2 ligands, such as, 19-kDa lipoprotein (LpqH), lipoproteins LprA (Rv1270) and LprG (Rv1411c). Lipomannan and phosphatidyl-*myo*-inositol mannoside (PIM) are also TLR-2 agonists, which interact with TLR-2 to initiate cellular activation. The 19-kDa lipoprotein (LpqH), secreted by MTB, was the first MTB ligand, which has been shown to interact specifically with TLR-2 to induce TNF-α and nitric oxide production in both murine and human macrophages. It is also a major inducer of interleukin-12 production in human monocytes [38]. Furthermore, it has been shown that TLR-2 is essential for the intracellular mycobacterial killing in macrophages, through the production of the antimicrobial peptide cathelicidin, an effect mediated by a vitamin-D related pathway [10]. However, studies in mice have shown that TLR-2 is not essential for host resistance against tuberculosis infection since the TLR-2-deficient mice were not compromised in their ability to induce Th1 immunity. On the contrary, it did exhibit exaggerated immuno-pathology. Additional *in vitro *studies have shown that engagement of TLR-2 with MTB ligands induces inhibition of macrophage MHC class-II antigen presentation [39] and also blocks macrophage responsiveness to IFN-*γ *[40,41]. These diverse data indicate that further studies are needed to elucidate whether TLR-2 is beneficial or harmful in humans in regard to tuberculosis.

The increased expression of Coronin-1, a protein that specifically inhibits the intracellular killing of live mycobacteria in mononuclear cells, is probably due to a specific subset of inactivated immature monocytes, which act as a Trojan horse for carrying mycobacteria from the inoculation sites to the other compartments of the cell body. However, although occult haematogenous dissemination is thought to occur following initial infection of humans with MTB, there is no direct evidence of this in the present study. The results observed here are rather consistent with recirculation of activated monocytes from the site of the infection.

This represents the well known process of haematogenous spread of tubercle bacilli during MTB infection via blood vessels and lymphatics, which involves the continuous turnover of macrophages between tuberculous lesions and the blood, a process that peaks during the phase of active infection [1,42]. Furthermore, during MTB infection, activated macrophages ingesting micro-organisms generate phagosomes that mature progressively along with the endocytic pathway, leading to fusion with late endosomes and ultimately to the formation of lysosomes [43,44]. As a result, the proper orchestration of these events leads to the destruction of the pathogen in phagolysosomes and the initiation of the appropriate innate immune response [45,46]. In striking contrast, the failure of phagosomes to fuse with lysosomes is a frequent finding following macrophage ingestion of *Mycobacterium tuberculosis *(MTB) and the variant *M. bovis *BCG, enabling these organisms to reside safely in vacuoles that support their survival and replication [31,32,47]. Since Coronin-1 is up-regulated in patients with tuberculosis infection (*i.e*. patients and LTBI close contact group) it is possible that Coronin-1 may have a role as a marker of MTB infection before the occurrence of the disease. Until now the diagnosis of tuberculous infection is based mostly on immunological tests such as the tuberculin skin test and the interferon gamma releasing assays (IGRAS). However, these tests are informative about the immunological status of an individual to mycobacterial antigens and are not helpful in distinguishing an active infection; therefore specific markers of the infection status are highly desirable [48,49].

Surprisingly, we have also observed an increased expression of Sp110 in the MTB infected groups (active TBC patients and LTBI groups) compared to non-infected closed contacts and the healthy population. However, three recently published molecular studies reported that there is no association between human pulmonary tuberculosis and Sp110 gene polymorphisms [50,51]. On the contrary, a previous study has reported an association of Sp110 polymorphisms with susceptibility to tuberculosis in West Africa [52]. The increased expression of Coronin-1 and Sp110 in the infected groups suggests that Sp110 may have a diverse role in humans compared to mice during MTB infection. It was recently reported that Sp110 expression promotes the replication of intracellular pathogens, as in *A. phagocytophilum*, an obligate intracellular tick-borne pathogen that causes human granulocytic anaplasmosis (HGA) in HL-60 cells, while silencing of Sp110 expression by RNA interference results in decreased levels of infection [53]. Thus, the role and function of Sp110 in human pulmonary tuberculosis needs further investigation.

In our study, we found a strong correlation between Coronin-1 and TLR-2 expression in nucleated blood cells of infected patients compared to the non-infected groups. Recent data point out a link between TLR-2 and calcineurin, which is the final effector molecule of phagosome-lysosome fusion in the Coronin-1 pathway. Activation of TLRs generally leads to nuclear translocation of the transcription factor NF-κB, a critical component within many proinflammatory pathways, [16] including those associated with chemokine gene expression [10]. Although TLRs share the ability to activate NF-κB, cross talk with other signalling pathways is one mechanism by which activation of specific TLRs may lead to different patterns of gene expression [35]. Another key signalling molecule of the immune response, calcineurin, has been reported to have a functional interaction with NF-κB [54]. In two recently published studies it has been suggested that calcineurin may play a predominant role in the mediation of TLR-2-stimulated chemokine responses in murine myoblasts and in human airway epithelial cells, [55,56] indicating that Coronin-1 is a down-stream molecule in the TLR-2 pathway cytokine activation. In this regard it is possible that TLR-2 signalling pathway mediates the Coronin-1 expression and thus facilitates the intacellular survival of *Mycobacterium tuberculosis *in human PBMCs. However, recent studies suggest a negative relation between TLR-2 and Coronin-1, because TLR-2 is essential for the transformation of the inactive vitamin-D_3 _to its active form and promotes the production of cathelicidin, which is a bactericidal substance for mycobacteria [10]. Furthermore, pre-treatment of human monocytes with vitamin- D_3_/retinoic acid, down-regulated the expression of Coronin-1 and inhibited the entry and intracellular survival of *M. tuberculosis *within the monocytes [57]. A recent report from Japan also suggested a functional counteraction between TLR-2 and Coronin-1 in macrophages infected with *Mycobacterium leprae *[58]. Thus, although the data in this report regarding the TLR-2 and Coronin-1 interplay in *M. tuberculosis *infection need further elucidation, there is actual evidence to support a possible positive and negative feedback functional relation between them.

The strong correlation between Sp110 with TLR-2 expression in PBMCs in infected patients compared to the non-infected groups suggests that Sp110 expression is mediated through a TLR-2 pathway. TLRs activation generally leads to nuclear translocation of the transcription factor NF-κB. Sp110 is a member of the nuclear body (NB) components that functions as a nuclear hormone receptor transcriptional co-activator [19]. Sp110 and other NB-associated proteins, induced by type I (α/β) and type II (γ) interferons (IFNs), play a role in IFN response and virus replication. Sp110 expression is induced in human peripheral blood leukocytes and spleen but not in other tissues [22]. These data indicate that the expression of Coronin-1 and Sp110 is probably related to a TLR-2 activation pathway. However, further studies are needed to clarify this assumption. A comparison with other well-known genes associated with immune activation (e.g. TNF) might better elucidate this hypothesis.

## Conclusions

In conclusion, in the present work we have shown that mRNA levels of Coronin-1, Sp110 and TLR-2 are increased in patients with tuberculosis and in close contacts of patients with latent tuberculosis infection. Although our patient cohort sample was not particularly large our findings suggest that these molecules are essential for the disease process. However, further studies are needed to investigate the clinical significance of these markers in *M. tuberculosis *infection.

## Competing interests

All authors of this paper declare that they have no financial or other potential conflicts of interest concerning the subject of this manuscript.

## Authors' contributions

PC conceived the idea and designed the study, made the initial analysis and interpretation of the results and has been involved in drafting and revising the manuscript. EF had a significant contribution in the molecular analysis of patient samples and approved the version to be published. NR had a significant contribution in the acquisition of patient data and has been involved in drafting and revising the manuscript. CG and AS had significant contribution in acquisition of patient data, and approved the final version of the article. SK had a significant contribution in acquisition and microbiological analysis of patient data, and has given final approval for the version to be published. AH made the statistical analysis and helped in the interpretation of the results. CR edited the manuscript and has given final approval of the version to be published. NP conceived the idea and designed the study. He had the main contribution in the interpretation of the results, drafted and revised the version of the manuscript to be published.

## Pre-publication history

The pre-publication history for this paper can be accessed here:

http://www.biomedcentral.com/1471-2334/10/243/prepub
